# Development of qPCR Detection Assay for Potato Pathogen *Pectobacterium atrosepticum* Based on a Unique Target Sequence

**DOI:** 10.3390/plants10020355

**Published:** 2021-02-13

**Authors:** Anna A. Lukianova, Peter V. Evseev, Alexander A. Stakheev, Irina B. Kotova, Sergey K. Zavriev, Alexander N. Ignatov, Konstantin A. Miroshnikov

**Affiliations:** 1 Shemyakin-Ovchinnikov Institute of Bioorganic Chemistry, Russian Academy of Sciences, 117997 Moscow, Russia; a.al.lukianova@gmail.com (A.A.L.); petevseev@gmail.com (P.V.E.); stakheev.aa@gmail.com (A.A.S.); szavriev@ibch.ru (S.K.Z.); 2 Department of Biology, Lomonosov Moscow State University, 119234 Moscow, Russia; kira1959@gmail.com; 3 Agro-Technical Institute, RUDN University, 117198 Moscow, Russia; an.ignatov@gmail.com

**Keywords:** *Pectobacterium atrosepticum*, qPCR, bacterial taxonomy, bacterial identification, sensitivity, soft rot, pathogen detection

## Abstract

The recent taxonomic diversification of bacterial genera *Pectobacterium* and *Dickeya*, which cause soft rot in plants, focuses attention on the need for improvement of existing methods for the detection and differentiation of these phytopathogens. This research presents a whole genome-based approach to the selection of marker sequences unique to particular species of *Pectobacterium*. The quantitative real-time PCR assay developed is selective in the context of all tested *Pectobacterium atrosepticum* strains and is able to detect fewer than 10^2^ copies of target DNA per reaction. The presence of plant DNA extract did not affect the sensitivity of the assay.

## 1. Introduction

Soft rot *Pectobacteriaceae* (SRP) cause blackleg and soft rot in potatoes (*Solanum tuberosum* L.), one of the most important food crops in the world. These diseases contribute substantially to crop damage, which results in considerable economic loss [1]. *Pectobacterium atrosepticum* (van Hall 1902, Gardan et al. 2003) (Pat), previously known as *Erwinia atroseptica* and *Pectobacterium carotovorum* subsp. *atrosepticum*, is an example of SRP and is recognized as being among the most significant bacterial pathogens of potatoes [2]. Pat aggressively colonizes the surface and the vascular system of potatoes, and in favorable conditions, the symptoms of the disease develop very quickly.

Despite the impaired ability of Pat to grow at higher temperatures [3,4], recent reports indicate that Pat can be a causative agent for potato soft rot in countries with a mostly hot climate, such as Egypt [5], Pakistan [6] and Indonesia [7]. Usually considered to be specific to the potato, Pat is nevertheless identified as a cause of bacterial diseases in sunflowers [8,9].

Currently, there is no reliable physical, chemical or biological method to control blackleg and soft rot under field conditions [10]. A modern practical approach is based on phytosanitary measures for the production and distribution of seed tubers. Given the currently changing phytopathological situation in the world [11], the spread of bacterial potato pathogens (especially aggressive ones like Pat) should be properly monitored.

A number of methods have been suggested for the identification and differentiation of SRP (reviewed in [12,13]). Pat has been detected by means of conventional PCR [14,15,16,17] and loop-mediated isothermal DNA amplification (LAMP) [18,19]. Most of these methods were developed using a limited number of available sequences of housekeeping genes, before the first complete genome of SRP was sequenced [20]. Currently, the NCBI GenBank contains more than 200 complete and draft genome sequences of SRP, and taxonomic distribution within genera *Pectobacterium* and *Dickeya* has become complex, comprising 29 species [21]. The polymorphism of 16 S RNA and other housekeeping genes of related species can be insufficient for reliable differentiation [22].

The most important part in developing taxon-specific primers is the search for sequences that would allow us to distinguish target organisms (“positive” group) from non-target organisms (“negative” group). A number of algorithms and pipelines have been developed to simplify the procedure of discriminatory primers search. These approaches can include alignment procedures [23,24,25] or an alignment-free strategy [26]. Each of these approaches has its advantages and disadvantages, relating to public availability, computation time and the facility to adjust parameters, but all of them have three key stages: (i) identification of common oligonucleotide sequences in target genomes, (ii) exclusion of these sequences if they are similar to non-target sequences and (iii) primer construction, usually using the Primer3 program.

The purpose of this work was to develop a specific and sensitive quantitative polymerase chain reaction (qPCR) assay for the rapid detection of Pat. It is based on species-specific primers constructed using the workflow developed in a user-friendly Geneious Prime environment.

## 2. Results

### 2.1. ANI Comparison and Phylogeny

**Figure 1:**
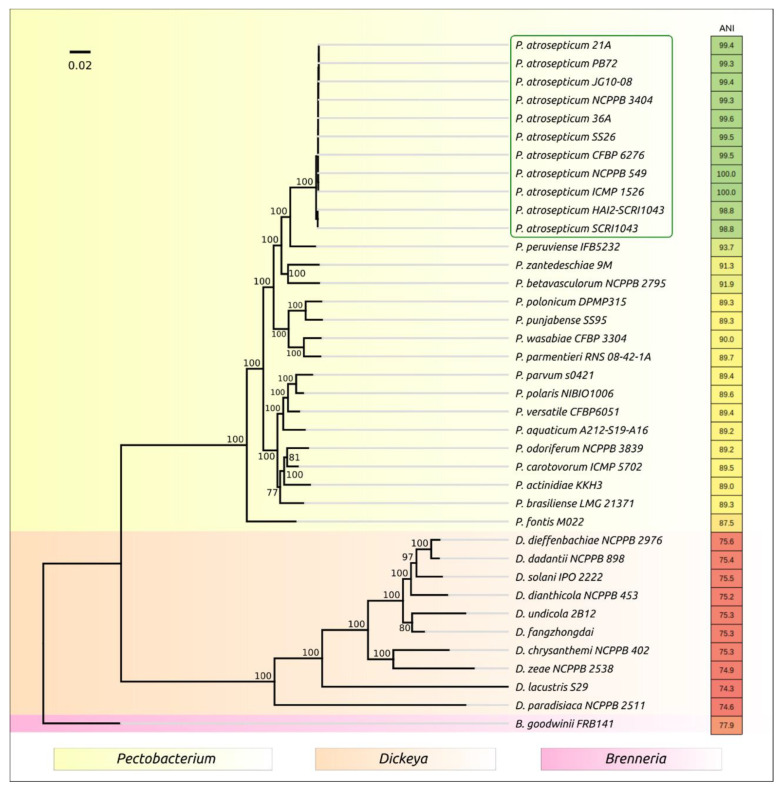
Best-scoring tree found by a maximum likelihood (ML) search with RAxML based on the 92 core genes concatenated nucleotide sequences. Gene sequences were extracted using the UBCG (up-to-date bacterial core gene) pipeline [27]. Bootstrap support values are shown above their branch as a percentage of 1000 replicates. The scale bar shows 0.02 estimated substitutions per site, and the tree was rooted to *Brenneria goodwini* FRB141. Average nucleotide identity (ANI) values compared to *P. atrosepticum* ICMP 1526 type strain were calculated with orthoANI [28] and are shown to the right of the organism name and colored according to a heat map scale, where a green color corresponds to the highest value and a red color corresponds to the lowest value.

As of mid-2020, the GenBank bacterial database contained 142 complete and draft genome sequences assigned to the *Pectobacterium* genus and 65 complete and draft genome sequences assigned to closely related SRPs of the *Dickeya* genus. The taxonomic affiliation of 25 genomes related to *Pectobacterium* and *Dickeya* remained unclear. Eleven genomes were attributed as *Pectobacterium atrosepticum* strains, including strains 21 A, 36 A, CFBP 6276, HAI2-SCRI1043, ICMP 1526 (type strain), JG10-08, NCPPB 549, NCPPB 3404, PB72, SCRI1043 and SS26. The average nucleotide identity (ANI) calculations for *Pectobacterium* strains grouped only 11 strains assigned to Pat in one cluster with 98.8% and higher ANI compared to the ICMP 1526 type strain (Figure 1).

Interestingly, the ANI of Pat strains varied in more narrow limits than some other *Pectobacterium* species, such as *P. brasiliense* and *P. versatile* [29], making the *P. atrosepticum* groups more homogeneous than clonal groups of other *Pectobacterium* species. The phylogeny constructed using concatenated housekeeping genes grouped Pat strains in a distinct clade and pointed to *P. peruviense* as the closest relative (Figure 1).

A BLAST analysis performed at mid-2020 and based on both complete and draft genome sequences retrieved from GenBank showed a high risk of false-positive results when using existing sets of Pat-specific primers. For instance, the primers offered by De Boer and Ward [14] are complimentary to the corresponding sequences in *P. peruviense* genomes potentially resulting in false-positive amplifications. The primers developed by Frechon et al. [15], Smid et al. [16] and Park et al. [17] can amplify target sequences in the genomes of *P. brasiliense*, *P. polaris* and *P. peruviense*, and conversely, are not completely complementary to the target sequences in the genomes of some Pat strains. This inconsistency is mostly explained by recent changes at the taxonomic level, assigning some previously sequenced strains to different species. Additionally, the phytopathological practice may not necessarily require fine differentiation of SRP because the symptoms of the disease are mostly similar for all species. However, the development of novel assays, characterized by improved specificity on the level of species, is still needed for both pathogen monitoring and taxonomic studies of this group of organisms.

### 2.2. Search for Species-Specific Primers

**Figure 2:**
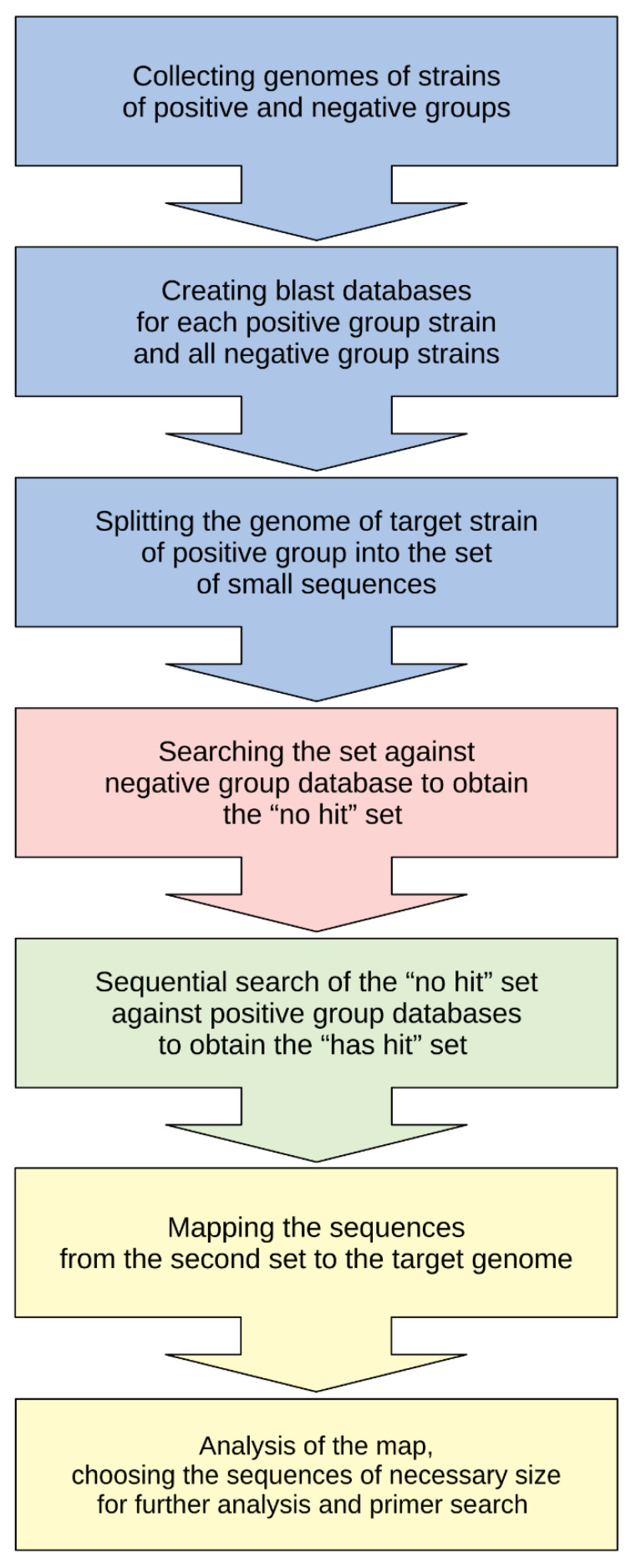
Flowchart of the search for species-specific sequences. The workflow includes three stages. Stage I is the preparation stage and consists of collecting the genomes, creating BLAST databases and splitting the target genome into sequences of 100 bases in length, overlapping by 90 bases, for performing BLAST searches in subsequent stages. Stage II is the search stage and consists of BLAST searches using negative and positive group databases to reveal possible species-specific regions. Stage III is the analysis stage and can include the analysis of the genome map, a BLAST search using other databases and choosing the most appropriate sequences for PCR purposes, and so on.

To identify the parts of the genome that were common to all Pat strains (positive group) but distinct from all other SRP genomes (negative group), a search workflow for such species-specific sequences was developed (Figure 2).

The workflow consisted of three stages. The first (preparation) stage entailed collecting genomes of positive and negative groups, creating BLAST databases for the collected sequences and splitting the target genome of the *P. atrosepticum* type strain into a set of short sequences of 100 bp, overlapping by 90 bp. The second stage began with a BLAST search of sliced sequences within a negative group database, to reveal ones which had no similarities with the negative group genomes. After that, the set of sliced sequences with no detected similarities in the genomes belonging to the negative group was sequentially searched against the BLAST databases constructed from the genomes of the positive group, to find sequences that had similarities with all the genomes belonging to the positive group. The third stage entailed mapping the sequences obtained from the second stage, which had no similarities with the negative group genomes but did have similarities with the positive group genomes, to the target genome, to analyze the regions in the target genome that were specific to the positive group.

Several genomic regions in which Pat could be distinguished from other SRPs were identified. These were checked, using a BLAST analysis on a custom database comprising *Dickeya* and *Pectobacterium* genome sequences and non-redundant NCBI nt database, to make sure that the sequences were absent in the genomes of other SRPs and host plants. The regions found contained both non-coding sequences and more than 20 genes (Supplementary Figure S1).

The primers and the probe (Table 1) were generated using Primer3Plus. Melting temperature of primers (60 °C for both), absence of hairpins and self-dimers were checked using the functions of Geneious Prime and by Primer Biosoft. A BLAST analysis was conducted of supposed species-specific genes with non-redundant NCBI nt database, and the analysis indicated that some genes had homologues in other bacteria not belonging to the genus of *Pectobacterium*. It is possible that these genes were acquired by horizontal transfer and significantly diverged from the moment of acquisition. The fact that the sequences found were conserved among all Pat strains can testify to their importance in the bacterial life cycle. Many species-specific sequences, which are present in Pat but absent in other SRP genomes, have diverged homologues among phages and plasmids and may be associated with the virulence factors and other genes beneficial to bacteria.

### 2.3. Conventional PCR

Designed primers were tested against a set of 38 strains of the laboratory collection. Detailed information on the origin of strains and methods of identification is presented in Supplementary Table S1. The set included five strains of *P. atrosepticum* (F004, F041, F048, F162, F163), six strains of *P. versatile* (F002, F016, F018, F035, F131, F135), four strains of *P. parmentieri* (F127, F148, F149, F174), three strains of *P. brasiliense* (F126, F152, F157), three strains of *P. polaris* (F109, F171, F182), one strain of *P. carotovorum* (F160), *P. aquaticus* (F164), *P. odoriferum* (F265) and *P. betavasculorum* (F258), as well as representatives of the genus *Dickeya: D. dianticola* (F077, F085, F117), *D. solani* (F012, F155) and *D. zeae* (F261). In addition, the set included eight insufficiently characterized strains that were isolated from rotting potatoes, which displayed pectolytic activity on the SVP medium but were not attributed to a particular species (F028, F034, F043, F061, F082, F097, F102, F105).

**Figure 3:**
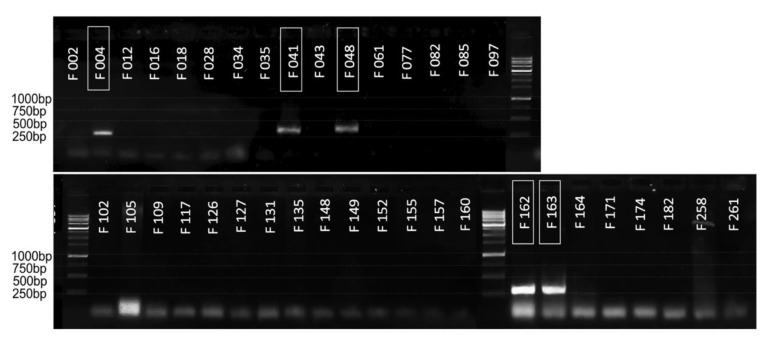
Specificity of the conventional PCR. *P. atrosepticum* strains are framed. “1 kb DNA Ladder” (Evrogen) was used to assess the amplicon size.

As shown in Figure 3, specific amplification of an expected 271 bp fragment occurred for all Pat strains (framed), while no amplification was observed for other strains.

### 2.4. Selectivity of qPCR Assay

To confirm the selectivity of detection, a number of qPCR reactions were performed with a large set of strains belonging to both SRP and pectolytic isolates of other genera and families.

Overall, 109 bacterial strains were used in this study, six of Pat, 50 strains of other representatives of the *Pectobacterium* genus and 15 strains of the *Dickeya* genus. The remaining strains were isolated from rotting potatoes and showed pectolytic activity, leaving pits on the SVP medium.

As shown in Supplementary Table S1, PCR analysis showed positive reactions with all strains of Pat that were used. Related species, such as *P. carotovorum*, *P. brasiliense*, *P. parmentieri, P. polaris, P. versatile, P. aquaticum*, *Dickeya* sp., other phytopathogenic bacteria and non-pathogenic soil enterobacteria did not show any amplification.

To confirm the absence of cross-amplification with potato DNA, reactions with DNA extracts of uninfected potatoes were performed as an additional control, which showed no amplification.

### 2.5. Sensitivity of qPCR Assay

**Figure 4:**
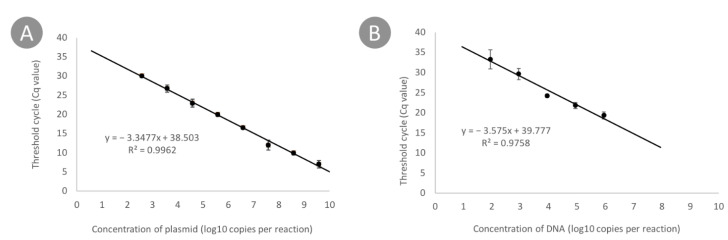
Standard curves showing the relationship between Cq and the quantity of plasmid DNA (**A**) genomic DNA of the *P. atrosepticum* SCRI1043 (**B**).

**Figure 5:**
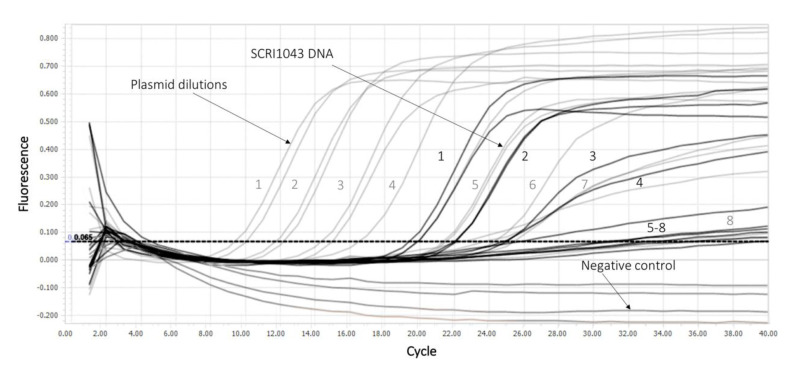
Sensitivity of *P. atrosepticum* detection using the PCR assay. Plasmid dilutions are marked in grey, genomic DNA dilutions are marked in black, the negative control is marked. The numbers represent the corresponding dilutions shown in Table 2.

Standard curves (Figure 4A,B) were generated by plotting the mean threshold cycle (Cq), which measured both a series of ten-fold dilutions of the positive control plasmid and the genomic DNA of Pat SCRI 1043, against the logarithmic concentration of DNA in each sample. An example of amplification curves is shown in Figure 5. The plasmid containing the target sequence was reliably detected in a concentration range from 10^9^ copies per reaction down to 10^2^ copies. The genomic DNA was detected in the range of 10^6^ copies to 87 per reaction (Table 2). The limit of detection (LoD) calculated on the basis of four repeated experiments on the construction of standard curves was 40 ± 10 copies per reaction, which corresponds to 10^3^ copies per mL.

All standard curves obtained were linear and had *R*^2^ values of 0.99 and 0.97 for plasmid and genomic DNA, respectively, and had a slope of −3.34 and −3.5, respectively. Hence, the corresponding PCR efficiencies were 0.99 and 0.93.

Field samples of potato infected with Pat were not available, because no serious outbreaks of blackleg or soft rot caused by Pat were registered in European Russia in the period 2019–2020. To prove that the presence of plant DNA extract in the reaction mixture does not lead to inhibition and cross-amplification during the reaction, the experiment was repeated with the addition of potato DNA extract to the reaction mixture. The approximate concentration of potato DNA per reaction was 25 ng. Based on the data obtained, the presence of potato DNA did not decrease the sensitivity of the assay. The potential presence of plant metabolites that may inhibit the reaction depends mostly on sample preparation and the DNA isolation technique. The adsorption-based kits (e.g., Promega Wizard Magnetic DNA purification recommended by Humphris et al. [13] or Proba-GS used in the current research) usually remove such impurities effectively.

The LoD in the presence of potato extract remained at 10^3^ copies/mL. Standard curves had a slope of 3.40 and 3.59 for plasmid and genomic DNA and had *R*^2^ values of 0.99. The PCR efficiency calculated based on the constructed standard curves was 0.96 and 0.89 for plasmid and genomic DNA, respectively (Supplementary Figure S2). This result is comparable to the data obtained in the absence of potato DNA extract. Thus, we propose that the assay can be used for the detection of Pat DNA in naturally infected potato samples.

### 2.6. Artificially Inoculated Tubers Testing

The samples of the skins of inoculated tubers of both tested varieties demonstrated a positive result (for Cq values see Table 3). Calculated concentration of Pat was approximately 10^5^ bacterial cells per mL of potato extract. This amount reflects the ability of the bacteria to adsorb to the potato skin and can be considered as insufficient to cause the soft rot symptoms of the undamaged tuber. However, this concentration of Pat applied to the wounded potato tissue resulted in the development of the disease.

## 3. Discussion

The approach to the search for species-specific sequences adopted in this research made it possible to find the regions of genomes that were able to discriminate between closely related species. The workflow in a user-friendly Geneious Prime environment was constructed for this purpose. This approach also enabled the employment of versatile methods beyond Primer3 for the generation of primers and made the visualization of species-specific sequences and primers fast and convenient. The approach could be applied not only for diagnostics purposes, but also for genome analysis and taxonomic studies [30].

The study found a significant number of potential species-specific sequences in *P. atrosepticum*. The selected species-specific sequence is present in all genomes of *P. atrosepticum* deposited in the NCBI GenBank and represents a fragment of the APC family permease gene (locus KCQ_RS03780 in the *P. atrosepticum* ICMP 1526^T^ genome). This gene, common to all strains of *P. atrosepticum*, does not belong to mobile elements, so it can be considered as a genomic feature conserved in all Pat strains. The species-specific genes found with the workflow encode proteins that are supposedly involved to metabolism, transport, DNA processing and ATP binding. Some of these proteins are hypothetical.

Preliminary tests showed that the APC gene was the most suitable for diagnostic purposes. This region has, therefore, been selected for further assays. The protein encoded by this gene belongs to the amino acid-polyamine-organocation (APC) superfamily of amino acid transporters found in all domains of life. This superfamily is one of the largest families of secondary transporters [31]. It is hypothesized that this ubiquitous protein [32] is a part of genomic islands in bacteria [33] and probably responsible for the translocation of the virulence factor in different organisms [34].

Optimized PCR conditions enabled the specific detection of the target sequence in all tested Pat samples, with both conventional PCR and the qPCR mode. No non-specific amplification or false-positive results were detected during the analysis of 109 bacterial strains, 73 of them belonging to the *Pectobacterium* or *Dickeya* genera. The demonstrated lack of inhibition and cross-amplification in the presence of potato DNA proves the suitability of this assay for detection in natural plant samples.

The assay has been shown to be sensitive enough to detect the pathogen at concentrations typically found in naturally infected tubers (10^2^–10^5^ cfu) and to detect concentrations below those considered to be sufficient for the development of symptomatic rot (10^6^ per tuber). Thus, this analysis is suitable for assessing the quality of potatoes and diagnosing the likely development of rot. The LoD calculated is comparable to the sensitivity characteristics of previously developed PCR assays, all of which returned a value of 10^2^–10^3^ cfu/mL [14,15,16,17]. We believe that the results of the experiment carried out on artificially inoculated tubers as well as the results of a sensitivity test performed in a presence of potato DNA extract suggest that the assay could be used for routine *Pat* detection.

PCR assays for Pat have been developed since the 1990s [14,15,16,17]. They are widely used for commercial and scientific purposes. However, a dramatic change in bacterial taxonomy led to a high risk of incorrect differentiation. Some *Pectobacterium* species are new and have been established as a result of thorough revision in taxonomy and phylogeny of SRP. Discriminating between these bacteria is a real challenge, as high genomic and physiological similarity between the species did not allow distinction by most of routine methods. As a result, it is difficult to find and test specific primer sets in a conventional way, testing them on strains of all presently known species of soft rot bacteria. The availability of ample genomic data makes it possible to develop an assay with improved specificity. The presented study is an example of using of a novel bioinformatic workflow for the development of highly sensitive and exceptionally specific assay for the detection of an emerging plant pathogen. The genome-based method we described here will be useful for fast development of soft rot bacteria and other pathogen groups by PCR diagnostic systems for further use in the seed certification programs for blackleg/soft rot-free planting material. We anticipate that the system will be further improved and applied to other target pathogens according to new taxonomical trends in plant pathology. 

## 4. Materials and Methods

### 4.1. ANI Calculations and Phylogeny

Bacterial genomes were downloaded from the NCBI GenBank bacterial database (ftp://ftp.ncbi.nlm.nih.gov/genbank (accessed on 23 November 2020)). A phylogenetic tree was generated by means of a UBCG pipeline, using 92 core genes [27]. The list of 92 genes used for the phylogenetic reconstruction included 43 ribosomal proteins, 9 genes of aminoacyl-tRNA synthetases, DNA processing and translation proteins and other conservative genes. To conduct bootstrap analysis phylogeny, the study team aligned concatenated sequences of 92 core genes made by UBCG with MAFFT (FFT-NS-x1000, 200 PAM/k = 2) and constructed bootstrap trees using the RAxML program (maximum likelihood method) [35,36] (GTR Gamma I DNA substitution model). The robustness of the trees was assessed by fast bootstrapping (1000). Average nucleotide identity (ANI) was computed using othoANI [28].

### 4.2. Species-Specific Sequence Search and Primer Design

Bacterial genomic sequences were cut using the EMBOSS splitter (http://emboss.sourceforge.net/apps/cvs/emboss/apps/splitter.html (accessed on 15 August 2020)). Custom databases were constructed using BLAST (https://blast.ncbi.nlm.nih.gov/Blast.cgi (accessed on 11 June 2020)). The set of split sequences was analyzed with BLAST, using a negative group database with settings “has hit/no hit” to obtain the “no hit” set. After this step, the “no hit” set was sequentially analyzed with BLAST using positive group databases with settings “has hit/no hit” to obtain the “has hit” set. The BLAST analyses were conducted in Geneious Prime 2019.1 (https://www.geneious.com (accessed on 11 March 2020)) with the settings: scoring 2–3, gap cost 5 2, word size 11 and E-value 10. The latter set was mapped to the target pseudochromosome, which was made of concatenated target genome contigs. Mapping sequences were conducted using the Geneious mapper with medium sensitivity settings. Primers and probe were generated with Primer3Plus (https://primer3.ut.ee/ (accessed on 22 September 2020)) and manually checked for the consistency of melting temperatures and for absence of hairpins and dimers formation using the functions of Geneious Prime and Primer Biosoft (http://www.premierbiosoft.com/NetPrimer/AnalyzePrimerServlet (accessed on 20 September 2020)).

### 4.3. Bacterial Strains, Growth Condition

The strains used in this study are the part of the local collection of the Laboratory of Molecular Bioengineering of the Institute of Bioorganic Chemistry, Russian Academy of Sciences. This collection with the entries numbered F contains above 300 type strains, sequenced strains and field isolates of bacteria associated with soft rot of potatoes. For details, see Supplementary Table S1. The strains were stored at −80 °C in 30% glycerol. Bacterial strains were grown at 28 °C in Lysogenic Broth (LB) medium.

### 4.4. DNA Isolation

Genomic DNA was isolated from bacterial strains using a Blood and Cell culture mini kit (Qiagen, Hilden, Germany), according to the manufacturer’s specifications.

Potato DNA was extracted using a “PROBA-GS” kit supplied by DNA-Technology LLC (Moscow, Russia), according to the manufacturer’s instructions.

Purified genomic DNA was quantified using a NanoProteometer N60 spectrophotometer (NanoProteometer, Munich, Germany). The same DNA samples diluted to 10 ng/μL were used for PCR and qPCR.

### 4.5. Polymerase Chain Reaction (PCR)

Conventional PCR was carried out in the final volume 25 µL using 5x Screen Mix (Evrogen, Moscow, Russia), 0.3 µM of each primer and 10 ng DNA per reaction. The reaction conditions were as follows: 94 °C for 300 s, then 30 cycles of 94 °C for 10 s, 65 °C for 10 s and 72 °C for 15 s. PCR products were separated with 1% agarose gel electrophoresis in TAE buffer and visualized using ethidium bromide. DNA Ladder 1 kb marker (Evrogen) was used for amplicon length estimation.

### 4.6. Construction of the Test Plasmid for Sensitivity Assay

Extracted DNA of *P. atrosepticum* strain SCRI1043 was used for further PCR amplification of the target sequence with designed primers. The reaction conditions were as in paragraph 2.5. The resulting PCR product was purified using ISOLATE II PCR and Gel Kit (Bioline, St. Petersburg, Russia) and cloned to pAL2-T vector (Quick-TA kit, Evrogen, Russia, Moscow). *Escherichia coli* Nova Blue strain (Novagen, Houston, TX, USA) was used for the propagation of the test plasmid. The insert was verified by Sanger sequencing using standard flanking primers (Evrogen).

### 4.7. qPCR

The qPCR assay was carried out in a LightCycler 96 (Roche, Basel, Switzerland). The amplification was performed in 35 μL volume containing 200 μM of each dNTP, 0.2 μM of probe, 0.35 μM of each primer and 2.5 μL (25 ng) of template DNA. Optimized thermocycling conditions were as follows: 94 °C for 300 s, then 45 cycles of 94 °C for 10 s, 60 °C for 10 s and 72 °C for 10 s. Each sample was analyzed in quadruplicate. Water was used as a negative control.

The exogenic internal plasmid-based control (IC) described earlier was used to exclude false negative results [37,38].

A sensitivity assay was performed on serial dilutions of the test plasmid and genomic DNA of the *P. atrosepticum* SCRI1043 strain.

Additionally, the experiment was repeated in the presence of potato DNA in the samples (25 ng of total DNA extracted from a potato tuber was added for each reaction) to simulate field sample testing and to confirm the absence of inhibition and cross-amplification with potato DNA.

The calculation of the plasmid and DNA copy number was carried out according to the following formula [39].

(1) $$\mathrm{Number}\;\mathrm{of}\;\mathrm{copies}=\;\frac{\mathrm{Amount}\left(\mathrm{ng}\right)\times N_{A}}{\mathrm{Lenght}\left(\mathrm{bp}\right)\times 10^{9}\times 660}$$

where N_A_ is Avogadro’s constant.

For all values, the standard deviation was calculated.

### 4.8. Artificial Inoculation of Potato Tubers

To assess the applicability of the designed assay to detect the population of Pat on the surface of the infected potato tubers, we used two major commercial varieties: Gala and Red Scarlett. Artificial inoculation of potato was performed according to methodology, described by Ranjan and Singh [40]. Then, the tubers were peeled, and the total DNA was isolated from the potato skins using a PROBA-GS kit (DNA-Technology LLC), according to the manufacturer’s instructions.

## Figures and Tables

**Table 1:** Species-specific sequences of generated primers and probe for *Pectobacterium atrosepticum* PCR detection.

**Primer F** 5′-CAGTAGGTTTGGGAGCAGGG
**Primer R** 5′-CCACTACCGATGATGCTCCC
**Probe** 5′-(6-FAM)-CGCGTCTTTTTT-(dT-BHQ-1)-GGGGTGTCGGCA-(Pi)
**Amplicon, 271 bp** CAGTAGGTTTGGGAGCAGGGTTAATGGCTGCAGTCTCTTATTTCCTTCTTCTTGCTGGTGTCGCGTCTTT TTTTGGGGTGTCGGCATCTGAGCTTATGAAAGGGATAACTGGAAGTTCATTACCCTGGTATGCCTATGC GCTAATTTGTTGGGCAGCGGTTGCATTACTGGGCTATTTGCATGTTGAACTTTCTGCAAAAGTATTGTC ATGGGTTATGCTTAGCGAAGTGATAATTTGTCTGGTTTTTTCTGGGAGCATCATCGGTAGTGG

**Table 2:** Mean C_q_ values for qPCR carried out on serial dilutions of genomic DNA of the *P. atrosepticum* SCRI1043 and corresponding plasmid.

	Plasmid DNA per Reaction			Genomic DNA per Reaction		
№	Plasmid Copies	Mean Cq	Standard Deviation	Genome Copies	Mean Cq	Standard Deviation
1	3.7 × 10^9^	7.03	0.96	8.7 × 10^5^	19.35	0.83
2	3.7 × 10^8^	9.98	0.54	8.7 × 10^4^	21.82	0.77
3	3.7 × 10^7^	12.02	1.28	8.7 × 10^3^	24.26	0.40
4	3.7 × 10^6^	16.58	0.35	8.7 × 10^2^	29.67	1.40
5	3.7 × 10^5^	20.01	0.51	87	33.3	2.40
6	3.7 × 10^4^	22.95	1.02	8.7	-	-
7	3.7 × 10^3^	26.75	0.95	0.87	-	-
8	3.7 × 10^2^	30.05	0.33	0.087	-	-

**Table 3:** Model experiment of Pat detection in potato samples.

№	Variety	Type	qPCR Assay		Pathogen Concentration, Copies of Pathogen DNA/mL of Potato Extract
			Cq Mean	Cq Error	
1	Red Scarlett	Neg. control	-	-	
2	Red Scarlett	Inoculated	28.46	0.56	5 × 10^5^
3	Gala	Neg. control	-	-	
4	Gala	Inoculated	29.55	0.95	3.6 × 10^5^

## Supplementary Materials

The following are available online at https://www.mdpi.com/2223-7747/10/2/355/s1, Supplementary Table S1: Selectivity of the qPCR method, Supplementary Figure S1, Supplementary Figure S2: Standard curves showing the relationship between Cq and the quantity of plasmid DNA genomic DNA of the *P. atrosepticum* SCRI1043 in the presence of potato DNA extract.
