# Supplementary material for: Development of qPCR Detection Assay for Potato Pathogen Pectobacterium atrosepticum Based on a Unique Target Sequence
Source: Plants (Basel). 2021 Feb 13;10(2):355. doi: 10.3390/plants10020355 (PMC7918688; doi:10.3390/plants10020355)
Supplement: Supplementary file 1 [file plants-10-00355-s001.zip › Supplementary Table 1 NEW.docx]

**Supplementary Table 1.** Selectivity of the qPCR method

| # | Strain designation | Alternative strain designation | Geographical region | Species | Genus/species determination | Mean  Cq | qPCR |
| --- | --- | --- | --- | --- | --- | --- | --- |
| 1 | F004 | PB72 | Moscow | Pat | NZ_PDDK00000000.1 | 21.35 | + |
| 2 | F041 |  | Moscow | Pat | 16S, PCR, MLST | 23.12 | + |
| 3 | F048 | 14 | Moscow | Pat | 16S, PCR, MLST | 18.80 | + |
| 4 | F162 | SCRI1043 | Scotland | Pat | NC_004547 | 16.28 | + |
| 5 | F163 | 21A | Belarus | Pat | NZ_CP009125 | 20,01 | + |
| 6 | F241 | 36A | Belarus | Pat | 16S, PCR, MLST | 21,13 | + |
| 7 | F164 | PB31 | Moscow | Paq | NZ_PJJA00000000.1 |  | - |
| 8 | F152 | PB29 | Moscow | Pbr | NZ_PJDM00000000.1 |  | - |
| 9 | F126 |  | Samara | Pbr | NZ_RRYQ00000000.1 |  | - |
| 10 | F128 |  | Samara | Pbr | 16S, PCR |  | - |
| 11 | F157 | PB38 | Moscow | Pbr | NZ_PJDL00000000.1 |  | - |
| 12 | F007 | 2892 | VKPM | Pwa | 16S, PCR, MLST |  | - |
| 13 | F127 | B5 | Kaluga | Ppa | 16S, PCR, MLST |  | - |
| 14 | F148 | PB20 | Moscow | Ppa | NZ_PDDJ00000000.1 |  | - |
| 15 | F149 | PB21 | Moscow | Ppa | 16S, PCR |  | - |
| 16 | F109 | 35 / E6 | Ethiopia | Ppo | NZ_RRYS00000000.1 |  | - |
| 17 | F171 | 2019.19-4 | Moscow | Ppo | 16S, MLST |  | - |
| 18 | F182 | 2019.41 | Moscow | Ppo | 16S, MLST |  | - |
| 19 | F002 | PB69 | Moscow | Pve | NZ_PDVY00000000.1 |  | - |
| 20 | F003 | PB70 | Moscow | Pve | NZ_PDVZ00000000.1 |  | - |
| 21 | F016 |  | Ryazan | Pve | NZ_RRYR00000000.1 |  | - |
| 22 | F018 |  | Moscow | Pve | NZ_PDVV00000000.1 |  | - |
| 23 | F131 | 12a | VIZR | Pve | NZ_PDVW00000000.1 |  | - |
| 24 | F135 | 2.1 / F5 | Moscow | Pve | NZ_PDVX00000000.1 |  | - |
| 25 | F160 | NCPPB312 | Denmark | Pca | NZ_JQHJ00000000.1 |  | - |
| 26 | F012 | Dfil | Voronezh | Dso | NZ_PGOJ00000.1 |  | - |
| 27 | D12 |  | Moscow | Dso | NZ_PGUT00000.1 |  | - |
| 28 | F085 | H3 | Moscow | Ddi | NZ_RSBK00000000.1 |  | - |
| 29 | F090 | 18b | Kaluga | Ddi | 16S, MLST |  | - |
| 30 | F008 | 3455 | VKPM | Pca | 16S, PCR |  | - |
| 31 | F053 | B6 | Unknown | Pca | 16S, PCR |  | - |
| 32 | F089 | G5 | Moscow | Pca | 16S, PCR |  | - |
| 33 | F118 | 522 / G2 | Moscow | Pca | 16S, PCR |  | - |
| 34 | F020 | 523 / I6 | Moscow | Pve | 16S, PCR |  | - |
| 35 | F021 | 526 / I4 | Moscow | Pve | 16S, PCR |  | - |
| 36 | F040 | 524 / H4 | Moscow | Pve | 16S, PCR |  | - |
| 37 | F172 |  | Novgorod | Pve | 16S, PCR |  | - |
| 38 | F034 | B9 | Kaluga | Pectobacterium sp. | 16S, PCR |  | - |
| 39 | F082 | H4 | Moscow | Dickeya sp*.* | 16S, PCR |  | - |
| 40 | F096 | F9 | Samara | Dickeya sp*.* | 16S, PCR |  | - |
| 41 | F153 | PB30 | Moscow | Lelliottia | NZ_PKFT00000000.1 |  | - |
| 42 | F154 | PB35 | Moscow | Lelliottia | NZ_PKFV00000000.1 |  | - |
| 43 | F159 | PB66 | Moscow | Lelliottia | NZ_PKFU00000000.1 |  | - |
| 44 | F113 |  | Unknown | Agrobacterium tumefaciens | 16S |  | - |
| 45 | F009 | 2220 | Moscow | Clavibacter michiganensis | 16S |  | - |
| 46 | F010 | 2222 | Unknown | Clavibacter michiganensis | 16S |  | - |
| 47 | F091 |  | Lithuania | Curtobacterium flaccumfaciens | 16S |  | - |
| 48 | F094 |  | Ehtiopia | Curtobacterium sp. | 16S |  | - |
| 49 | F065 | 442 / H8 | Moscow | Serratia sp. | 16S |  | - |
| 50 | F036 |  | Unknown | Stenotrophomonas sp. | 16S |  | - |
| 51 | F037 | 20/I3 | Moscow | Stenotrophomonas sp. | 16S |  | - |
| 52 | F038 | 1808v | Novgorod | Stenotrophomonas sp. | 16S |  | - |
| 53 | F045 | A3 | Krasnodar | Stenotrophomonas sp. | 16S |  | - |
| 54 | F049 | 1808b | Novgorod | Stenotrophomonas sp. | 16S |  | - |
| 55 | F055 |  | Moscow | Stenotrophomonas sp. | 16S |  | - |
| 56 | F060 |  | Ethiopia | Stenotrophomonas sp. | 16S |  | - |
| 57 | F066 |  | Samara | Stenotrophomonas sp. | 16S |  | - |
| 58 | F079 | 1860b | Voronezh | Stenotrophomonas sp. | 16S |  | - |
| 59 | F059 | 2354 | Unknown | Stenotrophomonas maltophilia | 16S |  | - |
| 60 | F039 | A8 | Lithuania | Pseudomonas sp. | 16S |  | - |
| 61 | F052 |  | Moscow | Pseudomonas sp. | 16S |  | - |
| 62 | F054 | E4 | Moscow | Pseudomonas sp. | 16S |  | - |
| 63 | F080 | C7 | Unknown | Pseudomonas sp. | 16S |  | - |
| 64 | F093 |  | Lithuania | Pseudomonas sp. | 16S |  | - |
| 65 | F114 |  | Unknown | Pseudomonas sp. | 16S |  | - |
| 66 | F115 | 1861a | Belgorod | Pseudomonas sp. | 16S |  | - |
| 67 | F123 |  | Moscow | Pseudomonas sp. | 16S |  | - |
| 68 | F125 |  | Unknown | Pseudomonas sp. | 16S |  | - |
| 69 | F013 | DD | Moscow | Xanthomonas sp. | 16S |  | - |
| 70 | F030 |  | Moscow | Xanthomonas sp. | 16S |  | - |
| 71 | F076 | C3 | Moscow | Xanthomonas sp. | 16S |  | - |
| 72 | F019 | I9 | Moscow | Advenella sp. | 16S |  | - |
| 73 | F024 | A1 | Moscow | Advenella sp. | 16S |  | - |
| 74 | F033 | 245/G8 | Moscow | Morganella sp. | 16S |  | - |
| 75 | F074 |  | Unknown | Morganella sp. | 16S |  | - |
| 76 | F015 | D6 | Moscow | Pca/Pve | PCR |  | - |
| 77 | F017 | 246 / I7 | Moscow | Pca/Pve | PCR |  | - |
| 78 | F023 | 2.4 / F1 | Tomsk | Pca/Pve | PCR |  | - |
| 79 | F025 | A7 | Lithuania | Pca/Pve | PCR |  | - |
| 80 | F027 | B9 | Moscow | Pca/Pve | PCR |  | - |
| 81 | F028 | D1 | Moscow | Pca/Pve | PCR |  | - |
| 82 | F047 | 10b | Moscow | Pca/Pve | PCR |  | - |
| 83 | F050 | 10a | Moscow | Pca/Pve | PCR |  | - |
| 84 | F058 | 512 / I1 | Moscow | Pca/Pve | PCR |  | - |
| 85 | F061 | 35 / F8 | Ethiopia | Pca/Pve | PCR |  | - |
| 86 | F062 | 246 / I8 | Moscow | Pca/Pve | PCR |  | - |
| 87 | F063 | D3 | Unknown | Pca/Pve | PCR |  | - |
| 88 | F064 | 520 / F6 | Moscow | Pca/Pve | PCR |  | - |
| 89 | F073 | 5 | Unknown | Pca/Pve | PCR |  | - |
| 90 | F077 | B6 | Ethiopia | Pca/Pve | PCR |  | - |
| 91 | F092 | E2 | Moscow | Pca/Pve | PCR |  | - |
| 92 | F098 | A4 | Moscow | Pca/Pve | PCR |  | - |
| 93 | F099 | 518 / E8 | Moscow | Pca/Pve | PCR |  | - |
| 94 | F111 | 301 / I8 | Moscow | Pca/Pve | PCR |  | - |
| 95 | F121 | C8 | Moscow | Pca/Pve | PCR |  | - |
| 96 | F124 | 2019.14 | Tver | Pca/Pve | PCR |  | - |
| 97 | F133 | C1 | Tyumen | Pca/Pve | PCR |  | - |
| 98 | F136 |  | Bryansk | Pca/Pve | PCR |  | - |
| 99 | F174 | 536 / I3 | Moscow | Pca/Pve | PCR |  | - |
| 100 | F035 | B7 | Kaluga | Pwa/Ppa | PCR |  | - |
| 101 | F014 | B1 | Tula | Ddi | PCR |  | - |
| 102 | F056 | A4 | Moscow | Ddi | PCR |  | - |
| 103 | F069 | C7 | Moscow | Ddi | PCR |  | - |
| 104 | F071 | C3 | Moscow | Ddi | PCR |  | - |
| 105 | F072 | B6 | Moscow | Ddi | PCR |  | - |
| 106 | F097 | 1913 / H9 | Moscow | Ddi | PCR |  | - |
| 107 | F117 | C9 | Bryansk | Ddi | PCR |  | - |
| 108 | F119 | F8 | Moscow | Ddi | PCR |  | - |
| 109 | F120 | C7 | Samara | Ddi | PCR |  | - |

+ Denotes positive PCR reaction

PCR methods used to verify the taxonomy of the isolates were the following: *Pectobacterium carortovorum* subsp*. carotovorum* (Pca) - [1], *Pectobacterium atrosepticum* (Pat) - [2], *Pectobacterium brasiliense* (Pbr) - [3], *Pectobacterium parmentieri* (former *P.wasabiae*) - [4], *Dickeya solani* (Dso) –[5], *Dickeya dianthicola* (Ddi) - [6].

These methods do not discriminate between *P.carotovorum* (Pca) and *P. versatile* (Pve) [7], and between *P .wasabiae* (Pwa) and *P. parmentieri* (Ppa). Therefore, the isolates characterized by PCR only are regarded as double designation.

Strains 1-29 represent characterized type strains with defined taxonomic attribution (genome sequencing or MLST)

Strains 30-40 are strains with taxonomic species/genus defined by 16S rDNA sequencing

Strains 41-75 are isolates of non-SRP bacteria associated with soft rot of potatoes and forming pits on CVP media [8]

Strains 76-109 are field isolates forming pits on CVP media and previously characterized by conventional PCR.

**References**

1. Kang, H. W.; Kwon, S. W.; Go, S. J. PCR-based specific and sensitive detection of Pectobacterium carotovorum ssp. carotovorum by primers generated from a URP-PCR fingerprinting-derived polymorphic band. *Plant Pathology* **2003**, *52*, 127–133, doi:10.1046/j.1365-3059.2003.00822.x.

2. De Boer, S. H.; Ward, L. J. PCR detection of Erwinia carotovora subsp atroseptica associated with potato tissue. *Phytopathology* **1995**, *85*, 854–858, doi:10.1094/Phyto-85-854.

3. Duarte, V.; De Boer, S. H.; Ward, L. J.; De Oliveira, A. M. R. Characterization of atypical Erwinia carotovora strains causing blackleg of potato in Brazil. *Journal of Applied Microbiology* **2004**, *96*, 535–545, doi:10.1111/j.1365-2672.2004.02173.x.

4. De Boer, S. H.; Li, X.; Ward, L. J. Pectobacterium spp. Associated with Bacterial Stem Rot Syndrome of Potato in Canada. *Phytopathology* **2012**, *102*, 937–947, doi:10.1094/PHYTO-04-12-0083-R.

5. van Vaerenbergh, J.; Baeyen, S.; de Vos, P.; Maes, M. Sequence diversity in the Dickeya flic gene: Phylogeny of the Dickeya genus and taqman® PCR for “D. solani”, new biovar 3 variant on potato in Europe. *PLoS ONE* **2012**, *7*, doi:10.1371/journal.pone.0035738.

6. Pritchard, L.; Humphris, S.; Saddler, G. S.; Parkinson, N. M.; Bertrand, V.; Elphinstone, J. G.; Toth, I. K. Detection of phytopathogens of the genus Dickeya using a PCR primer prediction pipeline for draft bacterial genome sequences. *Plant Pathology* **2013**, *62*, 587–596, doi:10.1111/j.1365-3059.2012.02678.x.

7. Portier, P.; Pédron, J.; Taghouti, G.; Fischer-Le Saux, M.; Caullireau, E.; Bertrand, C.; Laurent, A.; Chawki, K.; Oulgazi, S.; Moumni, M.; Andrivon, D.; Dutrieux, C.; Faure, D.; Hélias, V.; Barny, M.-A. Elevation of Pectobacterium carotovorum subsp. odoriferum to species level as Pectobacterium odoriferum sp. nov., proposal of Pectobacterium brasiliense sp. nov. and Pectobacterium actinidiae sp. nov., emended description of Pectobacterium carotovorum and description of Pectobacterium versatile sp. nov., isolated from streams and symptoms on diverse plants. *International journal of systematic and evolutionary microbiology* **2019**, doi:10.1099/ijsem.0.003611.

8. Hyman, L. J.; Sullivan, L.; Toth, I. K.; Perombelon, M. C. M. Modified crystal violet pectate medium (CVP) based on a new polypectate source (Slendid) for the detection and isolation of soft rot erwinias. *Potato Research* **2001**, *44*, 265–270, doi:10.1007/BF02357904.
