# Supplementary figures and images for: Development of qPCR Detection Assay for Potato Pathogen Pectobacterium atrosepticum Based on a Unique Target Sequence
Source: Plants (Basel). 2021 Feb 13;10(2):355. doi: 10.3390/plants10020355 (PMC7918688; doi:10.3390/plants10020355)

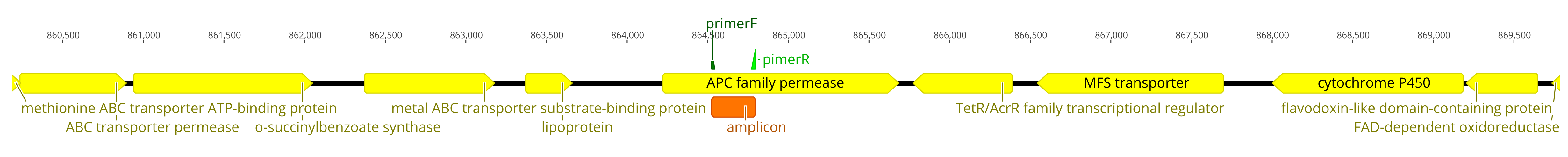

Supplement: Supplementary file 1 [file plants-10-00355-s001.zip › Suppl.Fig_1.png]

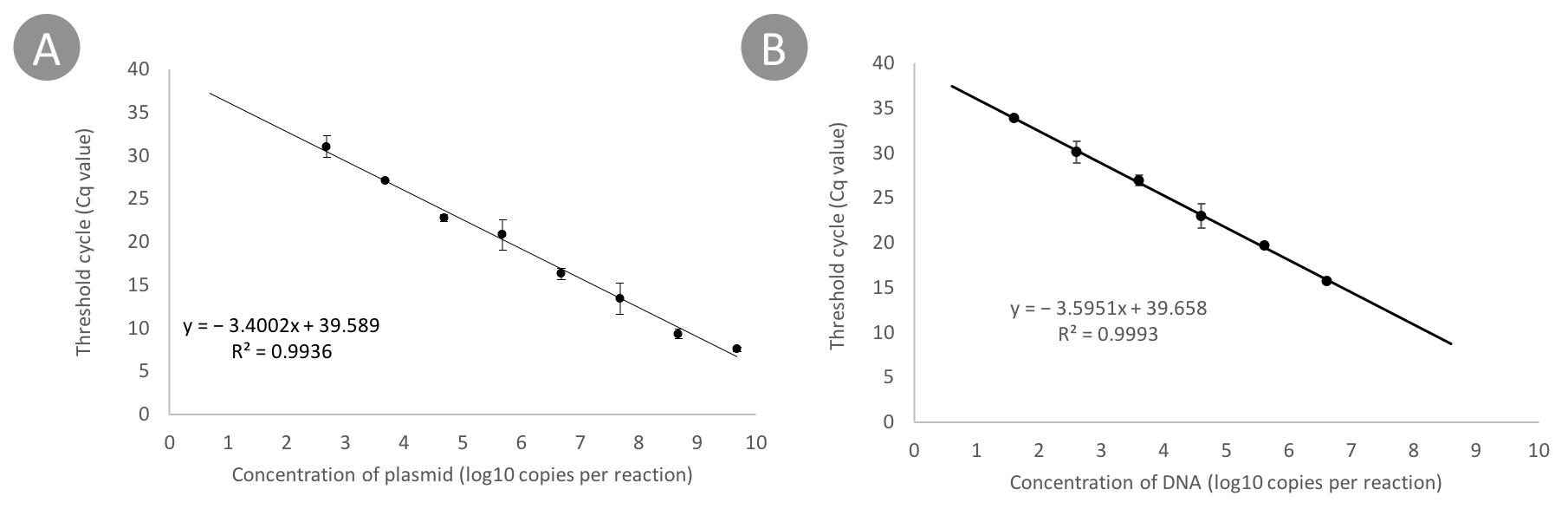

Supplement: Supplementary file 1 [file plants-10-00355-s001.zip › Supl_Fig_2.png]
